# Characterization and correlation analysis of oral NET markers and inflammatory factor levels in patients after orthodontic treatment: a pilot study

**DOI:** 10.3389/fimmu.2025.1490637

**Published:** 2025-05-06

**Authors:** Qian Liu, Axian Wang, Donghui Guo, Houzhuo Luo, Shishu Fang, Zhixin Song, Yi Wen, Fang Jin

**Affiliations:** ^1^ State Key Laboratory of Military Stomatology and National Clinical Research Center for Oral Diseases and Shaanxi Clinical Research Center for Oral Diseases, Department of Orthodontics, School of Stomatology, Air Force Medical University, Xi’an, China; ^2^ Department of Stomatology, General Hospital of Southern Theater Command of the Chinese People’s Liberation Army, Guangzhou, China

**Keywords:** orthodontic treatment, gingival crevicular fluid, saliva, neutrophil extracellular traps, inflammatory factor

## Abstract

**Introduction:**

Changes in oral neutrophil number and function may occur in patients after orthodontic treatment, affecting the oral immune microenvironment. However, the specific mechanisms are unclear. In this study, we describe the changes in the levels of neutrophil extracellular traps (NET) markers and inflammatory factors in the gingival crevicular fluid (GCF) and saliva of patients after orthodontic treatment and further explore the correlation between them.

**Methods:**

68 patients underwent fixed orthodontic treatment in the Department of Orthodontics from January 2021 to June 2023 were selected. GCF and saliva samples were collected from the patients 1 day before orthodontic treatment and 2 h, 24 h, and 1 week after orthodontic treatment to evaluate changes in NET marker and inflammatory factors. The differences in and associations between NET markers and inflammatory cytokine levels in the GCF and saliva of patients were evaluated.

**Results:**

After fixed orthodontic treatment, the neutrophil elastase (NE), myeloperoxidase (MPO), citrullinated histone 3 (CitH3), and MPO-DNA in the GCF and saliva of the patients increased gradually, the interleukin (IL)-1β and IL-8 in the GCF increased gradually, and there were significant differences among the different time points (P<0.05). There was a positive correlation between the NE, MPO, CitH3, MPO-DNA, IL-1β and IL-8 in the GCF of patients at 2 hours and 24 hours after orthodontic treatment (P<0.05). There was a significant positive correlation between the GCF and saliva levels of NE, MPO, CitH3, MPO-DNA, IL-1β and IL-8 (P<0.05); however, there was no statistically sex- or age-dependent differences in the NE, MPO, CitH3, MPO-DNA, IL-1β and IL-8 levels in the GCF of orthodontic patients (P>0.05).

**Conclusion:**

This study significantly reveals that NET marker levels in the GCF and saliva rapidly change following the initial orthodontic arch wire stress. The inflammation in periodontal tissues induced by orthodontic stress has the potential to trigger oral inflammation via the GCF. These findings are crucial for understanding the oral immune microenvironment changes during orthodontic treatment, providing a theoretical basis for preventing and treating orthodontic - related periodontal complications, thus having important implications for improving orthodontic treatment outcomes.

## 1 Introduction

Periodontal tissues undergo remodeling during orthodontic treatment, and the changes depend on the magnitude, direction, and duration of the applied force. Bone remodeling of periodontal tissues after orthodontic treatment is mediated by an inflammatory response characterized by vascular changes and leukocyte infiltration (1). This process may affect oral immune cell activity and cytokine expression levels, leading to alterations in the oral immune microenvironment, which in turn may promote the dysbiosis of oral flora and the development of periodontal tissue disease (2). Throughout orthodontic treatment, orthodontic appliances apply forces to teeth and periodontal tissues. These forces can trigger an inflammatory reaction in the periodontal tissues, affecting the overall oral immune environment. Cytokines and chemokines play important roles in this process, in which cytokines can regulate the activity of periodontal tissue cells, such as promoting the proliferation and differentiation of osteoblasts and osteoclasts, which are closely related to orthodontic tooth movement (3). Chemokines can attract immune cells to the site of inflammation, participating in the regulation of the oral inflammatory response (4). Therefore, it is essential to monitor these changes to assess the effectiveness of orthodontic treatment.

Gingival crevicular fluid (GCF) is a fluid produced by the gingival tissues, containing a variety of biochemical components like cytokines, enzymes, antibodies, and proteins. These elements found in GCF can indicate the inflammatory condition and immune response of the periodontal tissues. By analyzing the biochemical composition of GCF, one can track how the periodontal tissues react to the orthodontic forces, thereby evaluating the treatment results (5). Saliva has a similar composition to GCF and can be utilized to evaluate the oral immune environment. Components in saliva, including immunoglobulins, antibodies, and cytokines, reflect the status of the oral immune system. A comparison of the components in GCF and saliva can offer a comprehensive view of how the periodontal tissues and oral immune environment respond to orthodontic treatment (6). Therefore, by examining the biochemical elements in GCF and saliva, a deeper insight into the response of periodontal tissues and the oral immune environment to orthodontic forces maybe gained, guiding clinical treatment effectively.

In the process of oral microenvironment changes caused by orthodontic treatments, neutrophils play an important role in immune defense through deformation and chemotaxis, phagocytosis, and release of the neutrophil extracellular traps (NETs) (7). NETs are specialized structures released by neutrophils during infection or inflammatory responses, composed of DNA, histones, granule proteins, and other proteins. Previous studies have suggested that NETs can capture and kill pathogenic microorganisms, such as bacteria and fungi, thereby protecting oral health (8). Simultaneously, NETs may participate in the repair and regeneration of oral tissues and play a significant role in various oral diseases, including periodontal disease and oral cancer (9). Previous studies have also shown that when periodontal tissue is stimulated by mechanical force, a large number of neutrophils are recruited to the tissue and activated, periodontal tissue cells are activated, and a variety of inflammatory mediators, such as interleukin (IL) and chemokines, are released (10–12). Among them, myeloperoxidase (MPO) is one of the main markers of NETs, and its level is directly related to the number and activity of neutrophils in periodontal tissue. Previous studies have also suggested that MPO in the GCF can reflect the degree of oral neutrophil infiltration and tissue inflammation (13). In addition, under the action of orthodontic force, neutrophils migrate to periodontal tissue and undergo exosmosis into the GCF or saliva. Although it has been reported that MPO activity in whole saliva and GCF increases in a time-dependent manner the initial stage of tooth movement induced by fixed orthodontic treatment, it is difficult to describe the changes in oral NET activity by using MPO alone (14).

## 2 Specific objectives

Therefore, by examining the biochemical elements in GCF and saliva, a deeper insight into the response of periodontal tissues and the oral immune environment to orthodontic forces maybe gained, guiding clinical treatment effectively. The aim of this study was to investigate the changes in NET markers and related inflammatory factors in the GCF and saliva before and after initial orthodontic stress and to explore whether there is a correlation between the changes in the above factors in the GCF and saliva. This study will provides a theoretical basis for further study of inflammatory changes in the oral immune microenvironment induced by orthodontic treatment.

## 3 Methods

### 3.1 Trial design

This study was designed as a single-arm, prospective clinical trial.

### 3.2 Participants

Patients with malocclusion who were treated at our hospital from January 2021 to June 2023 were enrolled in this study. The inclusion criteria were as follows: ① patients with permanent dentition without a history of orthodontic treatment;② patients with a nonextraction treatment plan who experienced mild to moderate crowding during the initial alignment stage; ③The patient’s oral condition was suitable, and the gingival index, gingival sulcus bleeding index, and plaque index results were all normal before treatment; ④Patients with permanent dentition that were aged 11 years and had suitable oral hygiene habits. The exclusion criteria were as follows: ① Patients that had less than 20 permanent teeth in their mouth (missing teeth including extracted orthodontic teeth); ② Patients that had primary dental damage and white spots on the tooth surface with caries; ③ Patients that had periodontal disease; ④ Patients that had oral mucosal disease or oral odor; ⑤ Patients that had severe oral diseases, hematological diseases, or immune system diseases; and ⑥ noncommittal or unconscious individuals. All patients signed informed consent forms. This study was approved by the hospital ethics committee.

### 3.3 Interventions

Orthodontic patients who underwent initial bonding of metal self-ligating braces were selected for sample collection. The samples were collected before and during the initial stage of treatment. A uniform 0.014 NiTi round wire with a bent end was used as the first arch wire.

GCF and saliva samples were collected from patients 1 day before treatment and 2 hours, 24 hours, and 1 week after orthodontic treatment. (1) For each patient, 4 test teeth were selected: the left maxillary central incisor (21), left mandibular first premolar (34), right maxillary first molar (16), and right mandibular central incisor (41). The GCF collected from 4 teeth was mixed and used as the GCF test sample for each patient. GCF sampling: To ensure the consistency and comparability of GCF samples, we adopted a standardized sampling method. Cotton swabs were used to isolate saliva, and after removing dental plaque with a probe, a uniform quality Whatman No. 3 filter paper was gently inserted into the periodontal pocket along the tooth surface at four locations (mesial buccal, mesial lingual, distal buccal, and distal lingual) of the test tooth. When resistance was reached, the paper was held in place for 30 seconds. This standardized operation was designed to ensure that the amount of GCF absorbed by the filter paper was relatively consistent. Although we did not use a periotron to measure the GCF volume, this method can control the sample - taking process to a certain extent. After removal, 50 μL of an LPBS solution was added, and the samples were soaked for 30 minutes. The sample was centrifuged at 10000 rpm in a microcentrifuge with a rotor radius of 5 cm for 15 minutes at 4°C to obtain the supernatant, which was the GCF sample. (2) Saliva sampling: Before each collection of saliva samples, the patient rinsed their mouth with pure water and instructed the subject to chew a piece of paraffin. The secreted whole saliva (5 mL) was added to a 50 mL Alcon tube, and the saliva sample was immediately centrifuged at 4°C at 2500 rpm in a microcentrifuge with a rotor radius of 5 cm for 15 minutes to obtain the supernatant.

## 4 Outcomes

### 4.1 NET markers quantitation

The primary metrics were changes in the expression levels of NET markers in the GCF and saliva were evaluated. Salivary neutrophil elastase (NE), MPO, citrullinated histone 3 (CitH3), and the MPO-DNA complex were detected using an enzyme-linked immunosorbent assay (ELISA) kit. All ELISA test kits were purchased from Aibokang (Shanghai) Trading Co., Ltd., and Shanghai Zhucai Biotechnology Co., Ltd., with the following item numbers: ab270204, ab119605, ZC-55174, and ZC56556. The detection process strictly followed the instructions of the reagent kit: the specific antibody solution was added to the microplate, and the processed saliva sample was added to the microplate coated with the antibody so that the target molecule could bind specifically to the antigen or antibody. Unbound substances were removed through multiple washing steps to reduce nonspecific background signals. Enzyme-labeled antibodies that specifically bind to the target molecule were added to form complexes with the target molecule in the sample. The cells were washed again to remove unbound enzyme-labeled antibodies. Appropriate substrates were added to catalyze substrate reactions with enzyme-labeled antibodies to produce measurable signals, and then appropriate reaction stopping agents were added to stop the enzymatic reactions. The optical density values generated by the enzymatic reactions were measured using a multifunctional ELISA reader, and the expression levels of NET markers in each sample were calculated through standard curves.

The secondary metrics were changes in the expression levels of inflammatory cytokines in the GCF and saliva.

### 4.2 Inflammatory cytokines quantitation

The expression levels of inflammatory cytokines such as IL-1β, IL-6, IL-8, C-C motif chemokine ligand (CCL) 2, CCL20, and C-X-C motif chemokine ligand 10 (CXCL10) in saliva were measured via ELISA. The specific detection method used was similar to that used for the detection of NET biomarkers, and the corresponding ELISA kits were purchased from Thermo Fisher Biotech Co., Ltd., item numbers: BMS224-2, KHC0061, BMS204-3, BMS281, EHCCL20, and KAC2361.

### 4.3 Sample size calculation

The sample size calculation was based on a single group survey study, with the main metric being the change in the MPO level in the GCF after orthodontic treatment according to previous studies. This variable is a continuous variable, and the parameters used for sample size calculation were as follows: ① overall mean =953.3; ② overall standard deviation =589.8; ③ difference in clinical significance δ=1711.7; ④ inspection level α=0.05; and ⑤ test efficacy (1-β)=0.9. According to the PASS2023 calculation, 75 patients needed to be included. Considering a 15% dropout rate, 86.3 patients, 86 patients after rounding, needed to be included in the study.

### 4.4 Interim analyses and stopping guidelines

Not applicable.

### 4.5 Statistical analysis

SPSS 27.0 was used to analyze the data, and all quantitative data were subjected to a Shapiro–Wilks normality test. Those that conformed to a normal distribution are represented by Mean ± Standard Deviation ( $\bar{\text{x}}$ ± s), and an independent sample t test was used for intergroup comparisons. Measurement data that did not conform to a normal distribution are represented by M (P_25_, P_75_). The Mann–Whitney U test (*Z* test) was used for intergroup comparisons, and repeated-measures ANOVA (*F* test) was used for intragroup comparisons at different time points. Correlations between GCF and NET biomarker levels and inflammatory cytokine levels in saliva at different time points were analyzed by Pearson or Spearman correlation analysis. P<0.05 indicated a statistically significant difference.

## 5 Results

### 5.1 Patient clinical information

**Figure 1:**
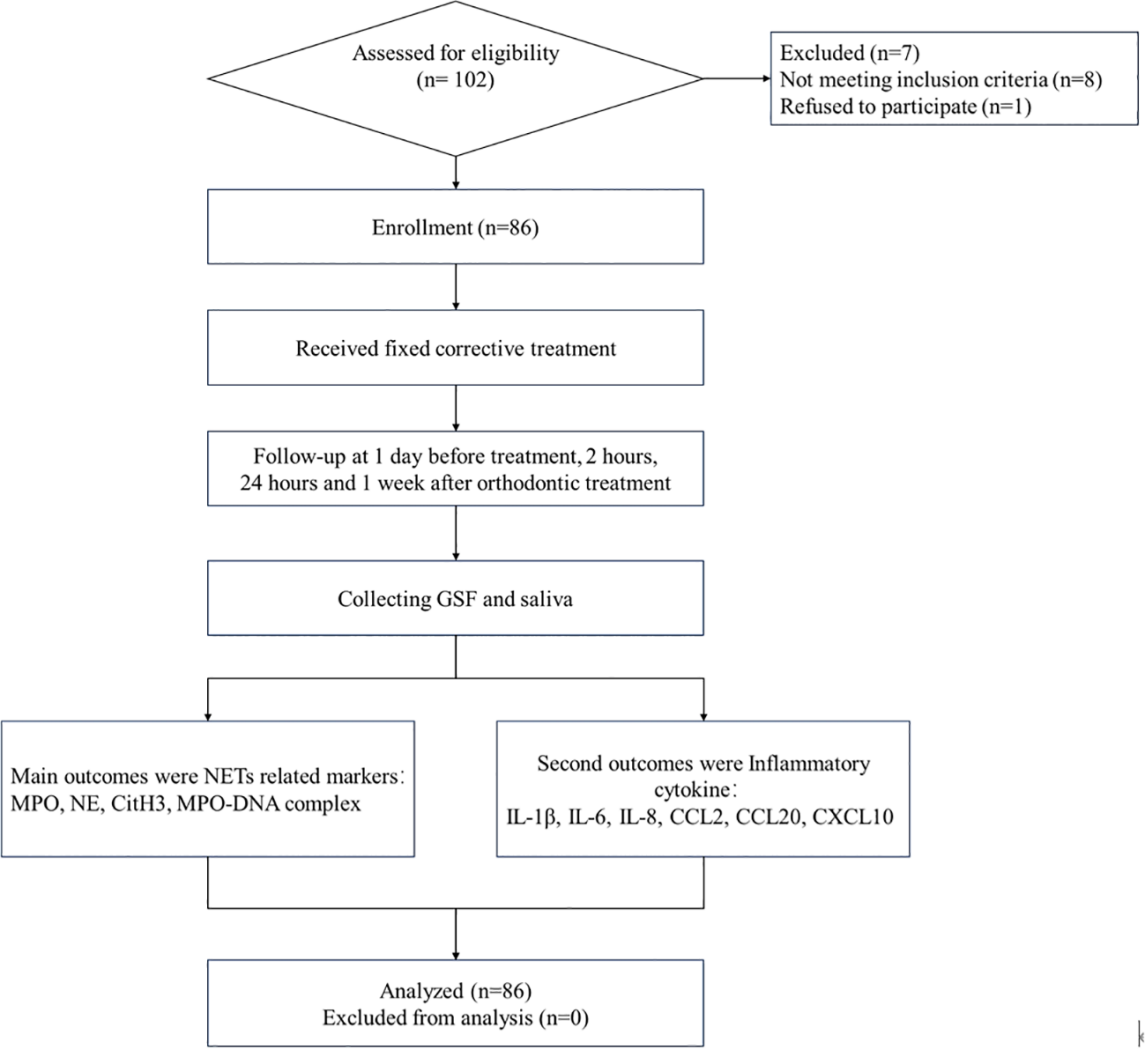
CONSORT flow chart showing patient flow during the trial.

The data of eighty-six patients were collected in January 2021, and the patients were followed up for the last time in June 2023. The average age of the enrolled patients was 20 (15, 26) years, ranging from 11 to 30 years. There were 32 male patients and 54 female patients. The average body mass index was 23.21 (21.92, 24.51) kg/m^2^, ranging from 19.18~28.02 kg/m^2^. The data are shown in **Figure 1**.

### 5.2 Variations in the levels of oral NET markers among individuals in the initial phase of orthodontic treatment

**Figure 2:**
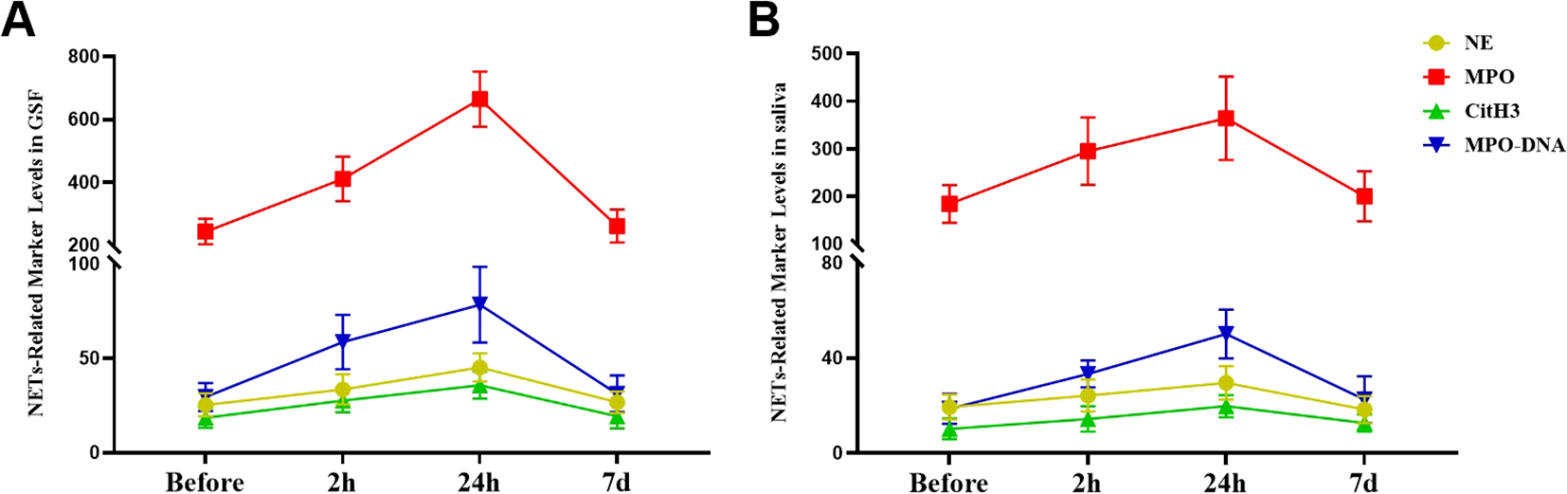
Alterations in the levels of NET biomarkers during the initial phase of fixed orthodontic treatment. **(A)** Variations in NET biomarker concentrations in the GCF at different time points after orthodontic treatment; **(B)** Fluctuations in the levels of NET markers in saliva at different time points after orthodontic treatment.

In the early stage of orthodontic treatment, as time increased, the levels of NE, MPO, CitH3, and MPODNA in the patient’s GCF and saliva gradually increased, and there were significant differences in the changes between the different time points (P<0.05, **Supplementary Tables S1**, **S2** in the **Supplementary Materials**). The trend of changes in NET markers in patient GCF and saliva over time is shown in **Figure 2**.

### 5.3 Variations in the levels of inflammatory cytokines during the initial phase of orthodontic treatment

**Figure 3:**
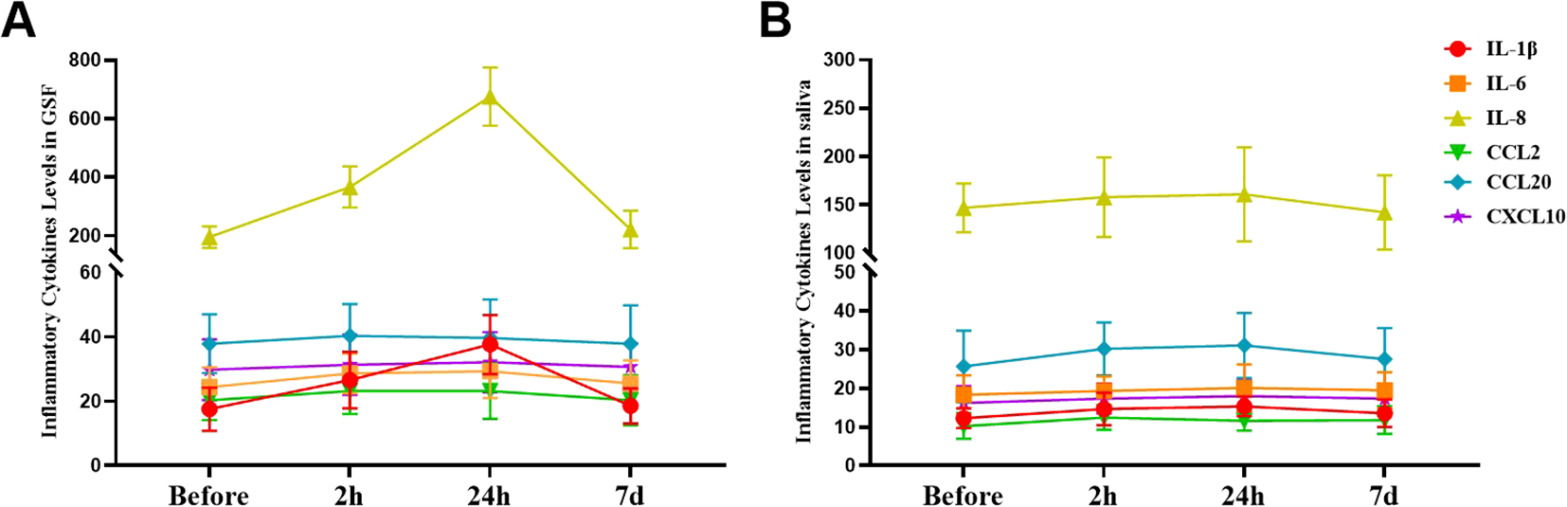
Alterations in the levels of inflammatory cytokines during the initial phase of fixed orthodontic treatment. **(A)** Variations in the levels of inflammatory cytokines in the GCF at different time points after orthodontic treatment; **(B)** Fluctuations in the levels of inflammatory cytokines in saliva at different time points after orthodontic treatment.

As the duration of orthodontic treatment increased, the levels of IL-1β, IL-6, and IL-8 in the GCF of patients gradually increased, but only the levels of IL-1β and IL-8 showed significant differences among the different time points (P<0.05, **Supplementary Table S3** in the **Supplementary Materials**). The levels of IL-1β, IL-6, and IL-8 in the saliva of patients gradually increased, but the differences among the different time points were not statistically significant (P>0.05, **Supplementary Table S4** in the **Supplementary Materials**). Regardless of whether they were in saliva or GCF, the levels of CCL2, CCL20, and CXCL10 in patients did not significantly change despite prolonged orthodontic treatment (P>0.05, **Figure 3**).

### 5.4 Association between the levels of inflammatory cytokines and NET markers in orthodontic patients

**Figure 4:**
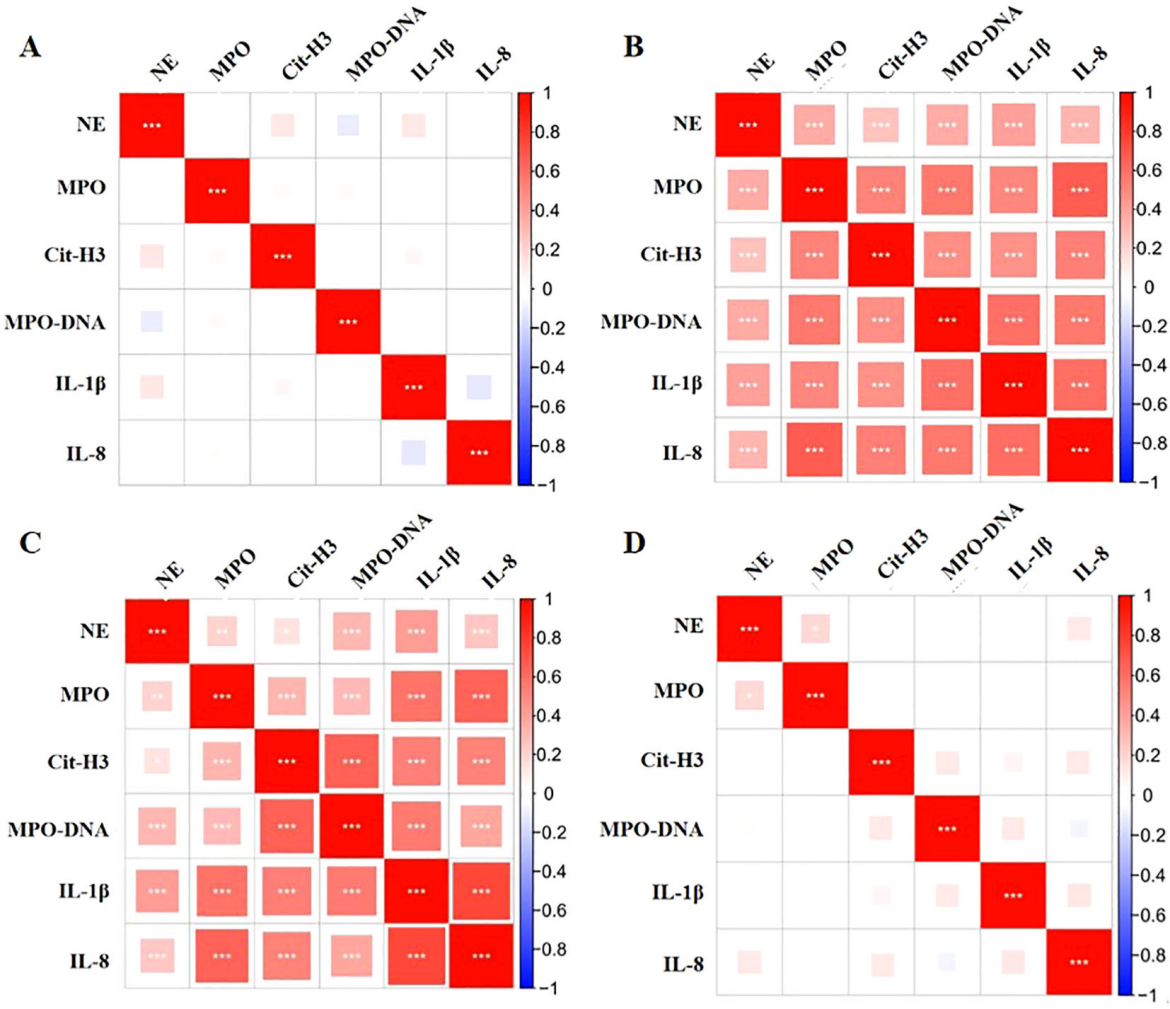
Correlations between oral inflammatory cytokines and NET markers in orthodontic patients. **(A-D)** Correlations between inflammatory cytokines and NET markers in the oral cavities of patients at various before treatment, and 2 hours, 24 hours, and 1 week posttreatment. *: P<0.05; **: P<0.01; ***: P<0.001

Spearman correlation analysis indicated that there was no statistically significant correlation between the levels of oral IL-1β and IL-8 or between the levels of NE, MPO, CitH3, or MPO-DNA in patients before and one week after they underwent orthodontic treatment (P>0.05, **Figures 4A, D**). The levels of IL-1β and IL-8 in the oral cavity were directly correlated with the levels of NE, MPO, CitH3, and MPO-DNA both at 2 and 24 hours after orthodontic treatment (P<0.05, **Figures 4B, C**).

### 5.5 Relationships between the levels of inflammatory cytokines and the levels of NETs in the GCF and saliva of orthodontic patients

Separately using the levels of IL-1β, IL-8, NE, MPO, CitH3, and MPO-DNA in GCF at different time points as independent variables, and the levels of IL-1β, IL-8, NE, MPO, CitH3, and MPO-DNA in saliva at different time points as dependent variables, Spearman correlation analysis indicated that there was no notable correlation between the levels of IL-1β, IL-8, NE, MPO, CitH3, or MPO-DNA in the saliva or GCF both prior to and one week after orthodontic treatment (P>0.05). At both the 2-hour and 24-hour time points following orthodontic treatment, a strong and positive relationship was observed between the concentrations of IL-1β, IL-8, NE, MPO, CitH3, and MPO-DNA in the saliva and GCF (P<0.05, **Supplementary Table S5** in the **Supplementary Materials**).

## 6 Discussion

Previous studies have shown that fixed orthodontic treatment not only leads to periodontal tissue remodeling and bone remodeling but also affects the function of periodontal and oral immune cells. Among them, neutrophils play a key role in maintaining homeostasis in the normal oral microenvironment. On the one hand, neutrophils may affect the immune microenvironment by migrating to the oral cavity and releasing inflammatory cytokines; on the other hand, they may affect the oral immune microenvironment by degranulation, phagocytosis and release of NETs (15, 16). Whether this induction of inflammation in the microenvironment is involved in periodontal complications such as periodontitis, gingival retraction, and disorders of the oral flora structure after orthodontic treatment remain to be studied. First, we urgently need to study whether NET function related to neutrophils in the oral immune microenvironment changes after orthodontic treatment.

MPO is an important component of NETs that has oxidative and antibacterial activity and can induce neutrophil aggregation and promote inflammation (17–19). Andrea et al. reported that the levels of GCF and saliva MPO in orthodontic patients increased significantly under different treatments, suggesting that neutrophils may be involved in changes in periodontal tissue and oral cavity inflammation after orthodontic treatment (20). In this study, we observed that the levels of GCF and MPO in saliva increased significantly within 2 hours and 24 hours after fixed orthodontic treatment. Combined with the results of Andrea et al., the concentration of MPO in the oral cavity and the changes that occur over time after orthodontic treatment have been more thoroughly described. In addition to MPO, NE, CitH3 and MPO-DNA complexes, the main components of NETs, are also involved in oral inflammation and immune homeostasis. Changes in NE, CitH3 and MPO-DNA levels may more comprehensively reflect changes in the characteristics of NETs (21). NE is an enzyme produced by neutrophils. Its main function is to degrade elastin in the extracellular matrix (22). Citrullination on histone H3 replaces arginine residues with CitH3, disrupting the positive charge interaction between histones and DNA. As a result, the chromatin structure becomes decondensed, allowing for the formation of NETs by loosening the densely packed chromatin (23). Additionally, MPO-DNA is a characteristic complex formed by MPO and free DNA released by neutrophils. It has oxidative and antibacterial activity and participates in the regulation of the immune response (24). In this study, we observed that the levels of NE, CitH3 and MPO-DNA increased significantly under short-term orthodontic treatment in both saliva and GCF, which was consistent with the change in MPO levels. The above conclusions strongly support the occurrence of neutrophil activation and NET release in the local cavity and whole oral cavity after orthodontic treatment. Considering that Wang et al. reported that NETs are closely related to the occurrence and development of delayed periodontitis and that Jiang et al. reported that NETs affect periodontal flora, we suggest that the change in saliva NET levels after orthodontic treatment may be a potential factor affecting the oral immune microenvironment and periodontal complications after orthodontic treatment (9, 25).

Numerous inflammatory mediators could also impact the oral immune microenvironment by directly influencing the microbiota or by triggering neutrophils’ involvement in the regulation process. Previous studies have suggested that interleukins may inhibit the degree of infection caused by pathogenic bacteria by promoting the aggregation and activation of inflammatory cells (26); moreover, IL-1β and IL-18 can also stimulate NET activation in neutrophils to cause changes in the immune microenvironment and accelerate the dysbiosis of oral flora (27–29). Chemokines such as CCL2 and CXCL10 also affect the composition and number of oral microflora through chemotaxis and activation of neutrophils, which in turn affect the oral immune microenvironment of orthodontic patients (30, 31). Our group also found that the expression of chemokines and ILs in the saliva of patients after orthodontic treatment was correlated with the oral inflammatory environment and the occurrence of dental caries and leukoplakia, which further suggested that the change in the inflammatory response in the oral cavity after orthodontic treatment may have a far-reaching effect on the composition of dental biofilm plaques (32). In this study, the levels of IL-1β, IL-6 and IL-8 in the GCF of orthodontic patients increased gradually, but the levels of IL-1β, IL-6 and IL-8 in the saliva did not change significantly, which was not consistent with the change in IL expression in the GCF. This may be attributed to the fact that the release of inflammatory factors is mainly limited to the areas that are in direct contact with the braces, while the changes in inflammatory cytokines released into saliva and the whole oral environment are not as dynamic as those in the cytokines released locally.

Is there any correlation between the immune inflammatory substances released by periodontal tissue into the GCF and the substances detected in the saliva? Through correlation analysis, we found that there was a significant positive correlation between the levels of NE, MPO, CitH3, and MPO-DNA in the GCF and saliva and between the levels of IL-1β and IL-8 in patients after orthodontic treatment, suggesting that the inflammatory factors in the periodontal tissue infiltrated into the GCF and saliva after orthodontic treatment (33). Based on the above results, we believe that neutrophils are quickly activated and release NE, CitH3, MPO, MPO-DNA complexes and other substances after orthodontic treatment, but the level of NE gradually returns to the preorthodontic treatment level as inflammation enters the chronic stage or when it is resolved. This finding is in line with previous studies on changes in NET markers and inflammatory factors in the oral cavity (34, 35). However, if oral NETs are continuously activated in orthodontic patients, the level of oral inflammation may be maintained at a high level, which may disrupt the homeostasis of the oral immune microenvironment. For example, the composition of the flora in the saliva changes and the levels of NET-related inflammatory factors increase in patients with postorthodontic treatment caries and leukoplakia. Therefore, we speculate that the change in the oral immune microenvironment after orthodontic treatment is an important factor affecting the biofilm structure and the dental flora; of course, this needs to be further investigated.

In addition, we divided the patients into groups according to age and sex and analyzed whether there were differences in the levels of NET markers and inflammatory factors in the GCF of different groups before and after orthodontic treatment to explore the impact of patient clinical characteristics on the above metrics. The results suggested that there was no significant difference in the levels of NET markers or inflammatory factors in the GCF of patients of different sexes and ages after orthodontic treatment, indicating that the changes in oral immune microenvironment caused by orthodontic treatment may be universal.

In summary, this study revealed that under the action of fixed orthodontic treatment, the expression levels of NET markers in the GCF and saliva changed in a short period of time and were strongly correlated. Changes in neutrophils, NET function and related inflammatory mediators in the early stage of orthodontic stress may play affect the oral immune microenvironment, and these changes may be reflected in the spread of periodontal inflammatory reactions into the GCF and the saliva. However, it should be noted that the causal mechanisms behind these changes remain unclear. Future research should focus on exploring whether orthodontic force directly triggers NETosis and what other inflammatory pathways are involved. By using molecular biology techniques such as RNA sequencing and protein interaction assays, we aim to clarify these mechanisms. This study not only was limited to observing the changes in NETs and inflammatory factors in the GCF but also analyzed the correlation between these factors in the GCF and saliva as a dynamic process, described the changes in the oral immune microenvironment after orthodontic treatment, and provided a new approach for explaining periodontal complications and oral flora changes after orthodontic treatment.

## 7 Limitations

This study has the following shortcomings: Firstly, in terms of sample selection, only patients who were wearing self-ligating braces were selected as the research subjects. In future studies, patients wearing different appliances should be included to expand the research scope. Secondly, the follow-up period of this study was only up to one week after orthodontic treatment. Given that orthodontic forces act for months or years, a longer follow-up is needed in future research to comprehensively understand the long - term changes of immune responses. Thirdly, this study lacks a control group (patients without orthodontic treatment). This makes it difficult to determine the exclusive influence of orthodontic stress on immune changes. Future research should include a proper control group to eliminate the interference of confounding factors such as oral hygiene, diet, and genetic predisposition. Fourthly, although this study identified correlations between NET markers and inflammatory factors, it did not explore the causal mechanisms. For example, it is unclear whether orthodontic force directly triggers NETosis or if other inflammatory pathways are involved. In future research, molecular studies such as RNA sequencing and protein interaction assays will be carried out to clarify the underlying mechanisms. In addition, we will increase the sample size in follow-up studies and conduct multicenter randomized controlled trials. Moreover, basic cytology, molecular biology and animal experiments will be carried out to explore the specific mechanism by which orthodontic treatment affects the oral immune microenvironment.

## Data Availability

The original contributions presented in the study are included in the article/**Supplementary Material**. Further inquiries can be directed to the corresponding author/s.

## References

[1] Pietro S Giampiero P. Staphylococcus aureus induces neutrophil extracellular traps (NETs) and neutralizes their bactericidal potential. Comput Struct Biotechnol J. (2021) 19:3451–7. doi: 10.1016/j.csbj.2021.06.012 PMC8220102 34194670

[2] Carlos S-R F. Zvi G Michael G Patrizia S. Neutrophil diversity in health and disease. Trends Immunol. (2019) 40:565–83. doi: 10.1016/j.it.2019.04.012 PMC7185435 31160207

[3] Akashi Y Nagasaki A Okawa H Matsumoto T Kondo T Yatani H. Cyclic pressure-induced cytokines from gingival fibroblasts stimulate osteoclast activity: Clinical implications for alveolar bone loss in denture wearers. J prosthodontic Res. (2023) 67:77–86. doi: 10.2186/jpr.JPR_D_21_00238 35185110

[4] Sahingur SE Yeudall WA. Chemokine function in periodontal disease and oral cavity cancer. Front Immunol. (2015) 6:214. doi: 10.3389/fimmu.2015.00214 25999952 PMC4419853

[5] Fatima T Khurshid Z Rehman A Imran E Srivastava KC Shrivastava D. Gingival crevicular fluid (GCF): A diagnostic tool for the detection of periodontal health and diseases. Molecules (Basel Switzerland). (2021) 26:1208. doi: 10.3390/molecules26051208 33668185 PMC7956529

[6] Mohideen K Chandrasekaran K Veeraraghavan H Faizee SH Dhungel S Ghosh S. Meta-analysis of assessment of total oxidative stress and total antioxidant capacity in patients with periodontitis. Dis Markers. (2023) 2023:9949047. doi: 10.1155/2023/9949047 37937148 PMC10627720

[7] Bushra A Hyeran Helen J Jia N Dongxu Q Alyssia L Julie J H. Osteoimmunology in periodontitis and orthodontic tooth movement. Curr Osteoporos Rep. (2023) 21:128–46. doi: 10.1007/s11914-023-00774-x PMC10696608 36862360

[8] Kim TS Moutsopoulos NM. Neutrophils and neutrophil extracellular traps in oral health and disease. Exp Mol Med. (2024) 56:1055–65. doi: 10.1038/s12276-024-01219-w PMC11148164 38689085

[9] Wang J Zhou Y Ren B Zou L He B Li M. The role of neutrophil extracellular traps in periodontitis. Front Cell infection Microbiol. (2021) 11:639144. doi: 10.3389/fcimb.2021.639144 PMC8012762 33816343

[10] C. Fernanda VS Paul K. Neutrophils and NETs in modulating acute and chronic inflammation. Blood. (2019) 133:2178–85. doi: 10.1182/blood-2018-11-844530 30898862

[11] Yingyi C Siyan L Lili W Yitong L Juan D Zhenhua L. Epigenetic regulation of chemokine (CC-motif) ligand 2 in inflammatory diseases. Cell Prolif. (2023) 56:e13428. doi: 10.1111/cpr.13428 36872292 PMC10334270

[12] Chuyi T Monowar A Ping W. The vitals of NETs. J Leukoc Biol. (2020) 110:797–808. doi: 10.1002/JLB.3RU0620-375R PMC9059135 33378572

[13] Nishio C Wazen R Kuroda S Moffatt P Nanci A. Disruption of periodontal integrity induces expression of apin by epithelial cell rests of Malassez. J periodontal Res. (2010) 45:709–13. doi: 10.1111/j.1600-0765.2010.01288.x 20572917

[14] Navarro-Palacios A García-López E Meza-Rios A Armendariz-Borunda J Sandoval-Rodríguez A. Myeloperoxidase enzymatic activity is increased in patients with different levels of dental crowding after initial orthodontic activation. Am J orthodontics dentofacial orthopedics: Off Publ Am Assoc Orthodontists its constituent societies Am Board Orthodontics. (2014) 146:92–7. doi: 10.1016/j.ajodo.2014.04.015 24975003

[15] Huixun D Juliet M B Sergei B Valentina A Wendy F L Daniel A W. Tuning immunity through tissue mechanotransduction. Nat Rev Immunol. (2022) 23:174–88. doi: 10.1038/s41577-022-00761-w PMC9379893 35974148

[16] Hao Z Yanghanzhao W Mengdi Q Wenqian L Dan W C. Juan P. Neutrophil, neutrophil extracellular traps and endothelial cell dysfunction in sepsis. Clin Transl Med. (2023) 13:e1170. doi: 10.1002/ctm2.1170 36629024 PMC9832433

[17] Asma F Kumar TA Abdul S Safiya S Arshad H. An evaluation and comparison of myeloperoxidase enzymatic activity during initial orthodontic alignment: an *in vivo* study. J Orthod. (2017) 44:169–73. doi: 10.1080/14653125.2017.1350329 28705081

[18] Kegong Z Wenchang H Xiaofeng G Ye Z Changxing H. Progress in mechanism of formation of neutrophil extracellular traps: Review. Xi Bao Yu Fen Zi Mian Yi Xue Za Zhi. (2020) 36:561–4. 32696748

[19] Pedro B Pavillard LE de la Torre-Torres R. Inflammasome and oral diseases. Exp Suppl. (2018) 108:153–76. doi: 10.1007/978-3-319-89390-7_7 30536171

[20] M. Andrea M A. Patricia AF L. Fernanda V G. Raquel F F. Jose TL. Myeloperoxidase activity is increased in gingival crevicular fluid and whole saliva after fixed orthodontic appliance activation. Am J orthodontics dentofacial orthopedics: Off Publ Am Assoc Orthodontists its constituent societies Am Board Orthodontics. (2010) 138:613–6. doi: 10.1016/j.ajodo.2010.01.029 21055602

[21] Andy W Kumar JR Jörgen E Doron A Anne Marie Lynge P Gordon P. A guide to medications inducing salivary gland dysfunction, xerostomia, and subjective sialorrhea: A systematic review sponsored by the world workshop on oral medicine VI. Drugs R D. (2016) 17:1–28. doi: 10.1007/s40268-016-0153-9 PMC5318321 27853957

[22] Voynow JA Meagan S. Neutrophil elastase and chronic lung disease. Biomolecules. (2021) 11:1065. doi: 10.3390/biom11081065 34439732 PMC8394930

[23] Yun-Rong C Xu-Dong X Fei S Bo-Wen X Mu-Yun Y Biao P. Simvastatin reduces NETosis to attenuate severe asthma by inhibiting PAD4 expression. Oxid Med Cell Longev. (2023) 2023:1493684. doi: 10.1155/2023/1493684 36778209 PMC9911252

[24] Remo P Mohamed S Gianni M D. Stephen R S. Guy W Gilda V. Neutrophil extracellular traps in asthma: friends or foes? Cells. (2022) 11:3521. doi: 10.3390/cells11213521 36359917 PMC9654069

[25] Qingsong J Yuxi Z Yusen S Xuedong Z Lei C Biao R. Interactions between neutrophils and periodontal pathogens in late-onset periodontitis. Front Cell infection Microbiol. (2021) 11. doi: 10.3389/fcimb.2021.627328 PMC7994856 33777839

[26] Hubert H Nahla I Johannes K Branislav Z Lisa-Marie M Lena H. ELISA detection of MPO-DNA complexes in human plasma is error-prone and yields limited information on neutrophil extracellular traps formed *in vivo* . PloS One. (2021) 16:e0250265. doi: 10.1371/journal.pone.0250265 33886636 PMC8062102

[27] Tianyu Z Wenzhou X Qiqi W Cong J Hongyan L Yang C. The effect of the “Oral-Gut” axis on periodontitis in inflammatory bowel disease: A review of microbe and immune mechanism associations. Front Cell infection Microbiol. (2023) 13. doi: 10.3389/fcimb.2023.1132420 PMC10008960 36923589

[28] Matsuo Y Marika S Hiroyuki I. The etiological consideration of oxidized low-density lipoprotein in periodontitis. J Oral Biosci. (2022) 65:19–23. doi: 10.1016/j.job.2022.09.006 36206991

[29] Jean-Christophe F Jean-François K Florence C D. Stephanie H Annick R Françoise B. Odontoblasts in the dental pulp immune response. J Exp Zool B Mol Dev Evol. (2008) 5:425–36. doi: 10.1002/jez.b.21259 19067439

[30] Ruoshui L F. NikoLaos G. Chemokines in cardiac fibrosis. Curr Opin Physiol. (2020) 19:80–91. doi: 10.1016/j.cophys.2020.10.004 PMC7665080 33195890

[31] Qian L Tao G Wei D Zhixin S Yi W Houzhuo L. Correlation between salivary cytokine profiles and white spot lesions in adolescent patients receiving clear aligner orthodontic treatment. BMC Oral Health. (2023) 23:857. doi: 10.1186/s12903-023-03561-3 37957648 PMC10641999

[32] Masaru Y Shinichi F. Is inflammation a friend or foe for orthodontic treatment?: inflammation in orthodontically induced inflammatory root resorption and accelerating tooth movement. Int J Mol Sci. (2021) 22:2388. doi: 10.3390/ijms22052388 33673606 PMC7957544

[33] Yulia K Simon G Ruiyuan Z Stephan B Holger W Matti A. The enzymatic and non-enzymatic function of myeloperoxidase (MPO) in inflammatory communication. Antioxidants (Basel). (2021) 10:562. doi: 10.3390/antiox10040562 33916434 PMC8066882

[34] Hai H Hongji Z O. Amblessed E Allan T. Neutrophil elastase and neutrophil extracellular traps in the tumor microenvironment. Adv Exp Med Biol. (2020) 1263:13–23. doi: 10.1007/978-3-030-44518-8_2 PMC11770835 32588320

[35] Baihong P A. Hasan B Wei C James M Baoling L Qiufang D. CitH3: a reliable blood biomarker for diagnosis and treatment of endotoxic shock. Sci Rep. (2017) 7:8972. doi: 10.1038/s41598-017-09337-4 28827548 PMC5567134

