# Supplementary material for: Characterization and correlation analysis of oral NET markers and inflammatory factor levels in patients after orthodontic treatment: a pilot study
Source: Front Immunol. 2025 May 6;16:1490637. doi: 10.3389/fimmu.2025.1490637 (PMC12089083; doi:10.3389/fimmu.2025.1490637)
Supplement: Supplementary file 1 [file DataSheet1.docx]

Table S1 Variations in NET markers in the GCF during the initial phase of orthodontic therapy (x̅±s)

| Index | Before ^a^ | 2h ^b^ | 24h ^c^ | 7d ^d^ | F/P |
| --- | --- | --- | --- | --- | --- |
| NE(ng/L) | 25.32±6.06 | 33.53±7.99 | 45.23±7.46 | 26.75±5.62 | 66.652/<0.001 |
| MPO(ng/L) | 243.36±40.64 | 411.35±70.91 | 664.57±87.68 | 261.36±52.54 | 340.597/<0.001 |
| CitH3(ng/L) | 18.57±5.38 | 27.69±6.33 | 35.79±7.19 | 19.32±6.32 | 31.035/<0.001 |
| MPO-DNA(ng/L) | 29.57±7.45 | 58.65±14.46 | 78.45±20.07 | 31.24±9.77 | 59.516/<0.001 |

Note: GCF: Gingival crevicular fluid; NE: Neutrophil elastase; MPO: Myeloperoxidase; CitH3: Citrullinated histone 3; ^a^Before the orthodontic treatment; ^b^2 hours after the orthodontic treatment; ^c^24 hours after the orthodontic treatment; ^d^7 days after the orthodontic treatment. The annotations in the following tables are consistent with the above.

Table S2 Variations in NET markers in saliva during the initial phase of orthodontic therapy (x̅±s)

| Index | Before ^a^ | 2h ^b^ | 24h ^c^ | 7d ^d^ | F/P |
| --- | --- | --- | --- | --- | --- |
| NE(ng/L) | 19.34±5.27 | 24.23±6.74 | 29.55±7.11 | 18.34±5.62 | 43.563/<0.001 |
| MPO(ng/L) | 184.34±39.78 | 295.23±70.91 | 364.33±87.68 | 200.45±52.54 | 156.58/<0.001 |
| CitH3(ng/L) | 10.23±4.32 | 14.35±5.36 | 19.78±4.67 | 12.65±3.56 | 23.67/<0.001 |
| MPO-DNA(ng/L) | 18.68±6.32 | 33.35±5.67 | 50.11±10.23 | 22.57±9.77 | 35.612/<0.001 |

Table S3 Variations in the levels of inflammatory cytokines in the GCF during the initial phase of orthodontic therapy (x̅±s)

| Index | Before ^a^ | 2h ^b^ | 24h ^c^ | 7d ^d^ | F/P |
| --- | --- | --- | --- | --- | --- |
| IL-1β(ng/L) | 17.57±6.70 | 26.57±8.78 | 37.65±9.17 | 18.56±5.45 | 44.220/<0.001 |
| IL-6(ng/L) | 24.36±6.17 | 28.66±6.15 | 29.33±8.35 | 25.56±7.17 | 3.805/0.053 |
| IL-8(ng/L) | 195.35±36.72 | 366.45±70.26 | 675.64±100.01 | 221.11±64.17 | 424.996/<0.001 |
| CCL2(ng/L) | 20.34±6.21 | 23.23±7.16 | 23.16±8.57 | 20.35±7.79 | 0.006/0.937 |
| CCL20(ng/L) | 37.89±9.16 | 40.34±9.91 | 39.67±11.98 | 37.89±11.91 | 0.040/0.841 |
| CXCL10(ng/L) | 29.78±9.48 | 31.34±9.32 | 32.11±9.35 | 30.67±7.34 | 1.879/0.172 |

Table S4 Variations in the levels of inflammatory cytokines in saliva during the initial phase of orthodontic therapy (x̅±s)

| Index | Before ^a^ | 2h ^b^ | 24h ^c^ | 7d ^d^ | F/P |
| --- | --- | --- | --- | --- | --- |
| IL-1β(ng/L) | 12.24±2.56 | 14.67±4.23 | 15.32±2.56 | 13.55±3.55 | 3.212/0.301 |
| IL-6(ng/L) | 18.34±5.01 | 19.32±3.78 | 20.12±6.12 | 19.52±4.67 | 3.332/0.395 |
| IL-8(ng/L) | 146.75±25.31 | 157.965±41.22 | 160.78±48.66 | 142.13±38.56 | 2.676/0.451 |
| CCL2(ng/L) | 10.23±3.3 | 12.45±3.21 | 11.58±2.56 | 11.79±3.56 | 0.106/0.789 |
| CCL20(ng/L) | 25.67±9.23 | 30.21±6.87 | 31.12±8.42 | 27.56±7.98 | 0.096/0.822 |
| CXCL10(ng/L) | 16.21±4.33 | 17.34±3.86 | 18.01±4.11 | 17.33±3.78 | 2.451/0.673 |

Table S5 Correlations between the levels of inflammatory cytokines and the levels of NET markers in the GCF and saliva of orthodontic patients

| Time point |  | IL-1β_GCF_ vs IL-1β_saliva_ | IL-8_GCF_ vs IL-8_saliva_ | NE_GCF_ vs NE_saliva_ | MPO_GCF_ vs MPO_saliva_ | CitH3_GCF_ vs CitH3_saliva_ | MPO-DNA_GCF_ vs MPO-DNA_saliva_ |
| --- | --- | --- | --- | --- | --- | --- | --- |
| Before ^a^ | *r* | 0.023 | 0.059 | 0.021 | 0.048 | 0.082 | 0.025 |
|  | *P* | 0.685 | 0.291 | 0.711 | 0.395 | 0.141 | 0.660 |
| 2h ^b^ | *r* | 0.395 | 0.385 | 0.443 | 0.420 | 0.399 | 0.446 |
|  | *P* | **<0.001** | **<0.001** | **<0.001** | **<0.001** | **<0.001** | **<0.001** |
| 24h ^c^ | *r* | 0.513 | 0.364 | 0.448 | 0.389 | 0.398 | 0.456 |
|  | *P* | **<0.001** | **<0.001** | **<0.001** | **<0.001** | **<0.001** | **<0.001** |
| 7d ^d^ | *r* | 0.015 | 0.007 | 0.076 | 0.023 | 0.098 | 0.063 |
|  | *P* | 0.792 | 0.894 | 0.175 | 0.677 | 0.078 | 0.262 |
